# Maximizing oyster-reef growth supports green infrastructure with accelerating sea-level rise

**DOI:** 10.1038/srep14785

**Published:** 2015-10-07

**Authors:** Justin T. Ridge, Antonio B. Rodriguez, F. Joel Fodrie, Niels L. Lindquist, Michelle C. Brodeur, Sara E. Coleman, Jonathan H. Grabowski, Ethan J. Theuerkauf

**Affiliations:** 1 Institute of Marine Sciences and Department of Marine Sciences, University of North Carolina at Chapel Hill, Morehead City, NC 28557, USA; 2 Marine Science Center, Northeastern University, 430 Nahant Road, Nahant, MA 01908, USA.

## Abstract

Within intertidal communities, aerial exposure (emergence during the tidal cycle) generates strong vertical zonation patterns with distinct growth boundaries regulated by physiological and external stressors. Forecasted accelerations in sea-level rise (SLR) will shift the position of these critical boundaries in ways we cannot yet fully predict, but landward migration will be impaired by coastal development, amplifying the importance of foundation species’ ability to maintain their position relative to rising sea levels via vertical growth. Here we show the effects of emergence on vertical oyster-reef growth by determining the conditions at which intertidal reefs thrive and the sharp boundaries where reefs fail, which shift with changes in sea level. We found that oyster reef growth is unimodal relative to emergence, with greatest growth rates occurring between 20–40% exposure, and zero-growth boundaries at 10% and 55% exposures. Notably, along the lower growth boundary (10%), increased rates of SLR would outpace reef accretion, thereby reducing the depth range of substrate suitable for reef maintenance and formation, and exacerbating habitat loss along developed shorelines. Our results identify where, within intertidal areas, constructed or natural oyster reefs will persist and function best as green infrastructure to enhance coastal resiliency under conditions of accelerating SLR.

Species distributions result from distinct regions of optimal fitness conditions, defined by critical boundaries regulated by physiological and external stressors^1^,^2^. As such, biological zonation is expressed both globally (e.g., latitudinal range limits) and locally, as in the intertidal zone for foundation species such as saltmarsh, mangrove and reef-forming bivalves. The forecasted acceleration in SLR^3^ will shift the position of critical boundaries in littoral systems. Thus, the resilience of sessile species confined to a narrow intertidal zone and their associated shorelines (both natural and developed) will be defined by the species’ ability to respond to moving boundary conditions. However, migration of coastal foundation species (e.g., oyster reefs and saltmarsh) will be hindered by shoreline development as part of the coastal squeeze^4^, resulting in reduced area suitable for colonization. Therefore, this anthropogenically-induced phenomenon accentuates the importance of self-maintaining accretional habitats that can match SLR^5^. Failure to maintain their position within the tidal range or migrate landward will result in replacement of biogenic reefs or marshes by unstructured habitats like sandflats^5^,^6^.

Intertidal habitats can provide disproportionately high levels of ecosystem services, such that coastal and estuarine ecosystems are among the most valuable on earth^7^,^8^,^9^,^10^,^11^,^12^. Unfortunately, continued population growth in coastal areas globally has led to the degradation of these ecosystems and reduced service delivery^11^,^13^,^14^, stimulating efforts to explore how these systems will respond to current and future anthropogenic stressors, such as accelerated SLR. As one of the only natural hard substrates along the Mid and South Atlantic Coast (USA), oyster reef habitat has been recognized as green infrastructure for shoreline protection^6^,^15^ and conservation of natural capital in the face of damaging storms and wave erosion^16^,^17^,^18^,^19^, even though they now occupy a small fraction of their distribution prior to massive harvesting during the last three centuries^20^,^21^,^22^. The effectiveness of using oyster reefs to enhance shoreline resiliency and reduce storm hazards along estuarine shorelines depends on understanding the biologically- and environmentally-driven thresholds separating oyster-reef production and growth from imminent degradation.

At whole-estuary scales, oyster growth responds to two overarching factors: salinity and aerial exposure, the amount of time intertidal oysters are exposed (or emerge) during a tidal cycle. Free-swimming oyster larvae require hard substrate to settle onto and typically grow on other oysters, which will form patches of unconsolidated oyster clusters that can eventually develop into large cohesive reef mounds (>1 km^2^) with densities exceeding 1000 individuals m^−2^ (refs 23,24). Historical observations^23^,^25^,^26^ of oyster abundances along estuarine gradients provide a foundational understanding of oyster response to varying salinity and aerial exposure. The euhaline (high salinity, between 30–35 psu) waters commonly found near coastal inlets are not conducive to subtidal oyster reef formation due to high levels of biotic stress on individual oysters from marine predators, competitors, bioeroders and pathogens^27^,^28^,^29^,^30^. However, mesohaline and polyhaline waters (moderate salinities, 5–18 and 18–30 psu respectively) offer oysters refuge from these marine stressors that are not tolerant of lower salinities, thereby allowing oyster reefs to persist subtidally unless they are exposed to hypoxic/anoxic (low oxygen) events^31^,^32^ or overharvesting^33^,^34^,^35^. The decimation to oyster populations as well as anthropogenic and SLR-driven changes to water quality have made restoration and sustainability difficult^36^,^37^,^38^, but there have been promising efforts^16^,^39^,^40^,^41^ that indicate restoration, recovery and sustainability are possible. Rates of oyster-reef growth appear comparable to rates of SLR^42^, and while intertidal oyster reefs have also exhibited the capacity for even greater growth^41^, it remains unclear as to which environmental conditions will provide the greatest return on investment from restoration efforts and ensure persistence with accelerations in SLR. Optimizing conservation and restoration efforts of oyster populations along our coasts requires a more precise understanding of how intertidal reefs grow in response to exposure-flooding cycles and forecasted SLR.

We investigated whole- and across-reef vertical growth, along with oyster density, on natural and constructed *Crassostrea virginica* (eastern oyster) reefs within the Rachel Carson National Estuarine Research Reserve, Back Sound, North Carolina (tidal range 0.92 m, salinity 30–35 psu). In total, 43 reefs provided spectrums of sizes (15–850 m^2^), ages (<1 − >100 years old), and tidal elevations (intertidal to subtidal) for our investigations. Constructed intertidal oyster reefs were created by forming dead oyster shells into 3 × 5 × 0.15 m piles in 1997, 2000, and 2011 that developed via natural oyster recruitment, growth and survivorship patterns^29^,^43^. We used a terrestrial laser scanner to measure variation in vertical growth across entire reefs constructed in 1997 and 2000, over a two-year time step (measured between 2010 and 2012, Fig. 1). Water-level data were collected within the study area in order to transform the reef elevations into the amount of time each portion of the reef spent emerged from the water (percent aerial exposure) during a tidal cycle (water level referenced to elevations using the North American Vertical Datum of 1988 [NAVD88]). We examined the relationship between reef growth and associated elevation to determine growth thresholds relative to an oyster reef’s position in the intertidal zone. Subsequently, we used those empirical data to develop a model that illustrates impacts of accelerating SLR on existing reefs and future reef construction as large-scale green infrastructure.

## Results and Discussion

**Figure 1:**
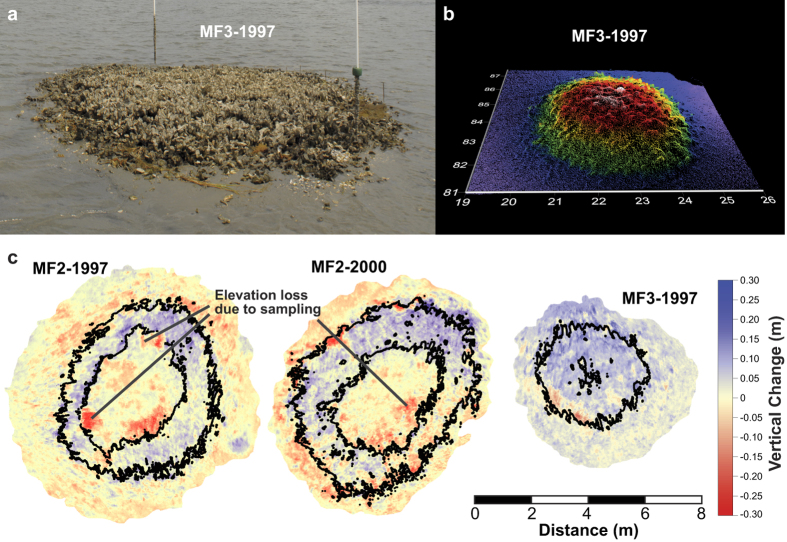
Measuring fine-scale growth on oyster reefs. (**a**) Photo and (**b**), oblique point cloud of oyster reef MF3-1997, both obtained in 2010 using a terrestrial laser scanner. (**c**), Digital elevation model subtraction maps of reefs constructed in 1997 and 2000. Reef scans were conducted in 2010 and 2012. Contour lines represent the 20% and 40% aerial exposure elevations in 2010.

**Figure 2:**
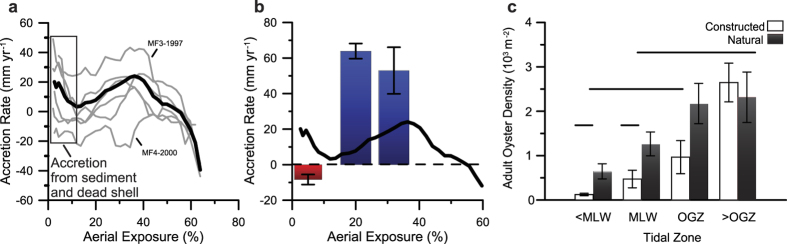
Analysis of oyster-reef growth and density over an aerial exposure gradient. (**a)** Mean vertical accretion rates by aerial exposure for decade-old constructed oyster reefs in Back Sound, North Carolina. Thick black line represents the mean vertical accretion rate from 2010 to 2012 for five of the decade-old reefs, excluding reef MF4-2000, which was heavily fished during the study period. (**b)** Bars represent the growth of newly constructed oyster reefs from 2011 (date of origin) to 2014 (mean ± standard error). Red and blue bars indicate loss and accretion respectively. Thick black line is the mean vertical accretion rate from the decade-old constructed reefs (from (**a)**). (**c)** Average adult oyster densities for natural (black) and constructed (white) reefs divided into four intertidal zones (mean ± standard error). The four zones include: below mean low water (<MLW), from MLW to 20% aerial exposure (MLW), the optimal-growth zone (OGZ, encompassing 20–40% aerial exposure), and above the OGZ (from 40% to approximately 60% aerial exposure). Tiered horizontal bars represent statistical similarity (α = 0.05).

Decade-old oyster reefs exhibited a unimodal relationship between average vertical-accretion rate and aerial exposure (Fig. 2a). Areas of highest mean growth were exposed 20–40% of the time, and this range represents an optimal-growth zone (OGZ). These reefs, along with natural oyster reefs, consistently exhibit a plateau morphology at 0.03 m NAVD88 (±0.05 m) (Supplementary Fig. 1), indicating that 55% (±1.5%) exposure is the upper zero-growth boundary (growth ceiling) for reefs in this region. The 10% exposure, occurring at −0.43 m NAVD88 in the Reserve, coincides with mean low water (MLW) and represents the lower zero-growth boundary for oyster reefs where accretional and erosional forces are balanced. Below 10% inundation, increases in accretion resulted from deposition of sediment and dead oyster shell at the reef edge. As an oyster reef is physically and biologically weathered^29^,^30^, material is transported from higher reef elevations downslope, mainly during periods of high wave and current energy, promoting lateral expansion. The lower portions of an oyster reef may experience increased vertical growth as the physical processes of sedimentation build those areas in to the OGZ. For example, a majority of reef MF3-1997 had just reached the center of the OGZ at the beginning of the study period and experienced the most vertical growth of all the decade-old oyster reefs (Figs 1 and 2a).

To verify these exposure boundaries, in 2011 we constructed oyster reefs along a gradient of sandflat exposures ranging from 0.01 to 18.0% (Supplementary Fig. 2 and Table 1) and average vertical reef growth was measured in 2014 using a Trimble 5800 GPS receiver (±1.5 cm vertical). The initial reef-top exposures ranged from 0.30 to 32.4%, which were comparable to the lower edge to mid-slope of natural, mature oyster-reef mounds located in the area. Those 3-year old reefs followed the same growth pattern as the decade-old reefs, with increasing aerial exposures resulting in greater reef growth rates, and little to no growth when those older reef were located below 10% exposure (Fig. 2b). Although the shallower reefs exhibited rapid vertical growth (4–8 cm yr^−1^), with the shallowest reef reaching 45% exposure at the end of the study period, our observation period was too short for these reefs to reach the growth ceiling and become confined by the stress of limited inundation. Reefs below MLW did not sustain growth, and anomalously-high accretion rates measured on some reefs were caused by migrating sand ripples converting the deep shell piles into sand mounds (Fig. 2b), as has been observed in other sandy environments^44^. While this overall growth pattern reinforces our results from decade-old reefs, it also indicates that newly-constructed oyster reefs have the potential to grow twice as fast as mature reefs. Thus, there likely is a progression of diminishing vertical growth from substrate colonization to reef maturation as the oyster reef approaches the growth ceiling and the area of the OGZ narrows to the reef flanks (Fig. 1c).

Oyster recruitment, growth and survival collectively mediate oyster reef accretion rates, and therefore, oyster density should generally be correlated with reef accretion. Adult oyster density in both natural and restored reefs also matched the observed reef growth pattern, except at the highest elevations of the reef (>OGZ), where density continues to increase as oysters recruit and fill the interstitial space (Fig. 2c) but are still limited in overall growth by desiccation stress (growth ceiling). Adult oyster densities were greater on natural reefs than restored reefs in all but the topmost region of the reefs (Fig. 2c). Very low adult oyster density below 10% exposure further supports our observation that reef accretion at the base (Fig. 2a) is from accumulation of sediment and shell material, not oyster growth.

**Figure 3:**
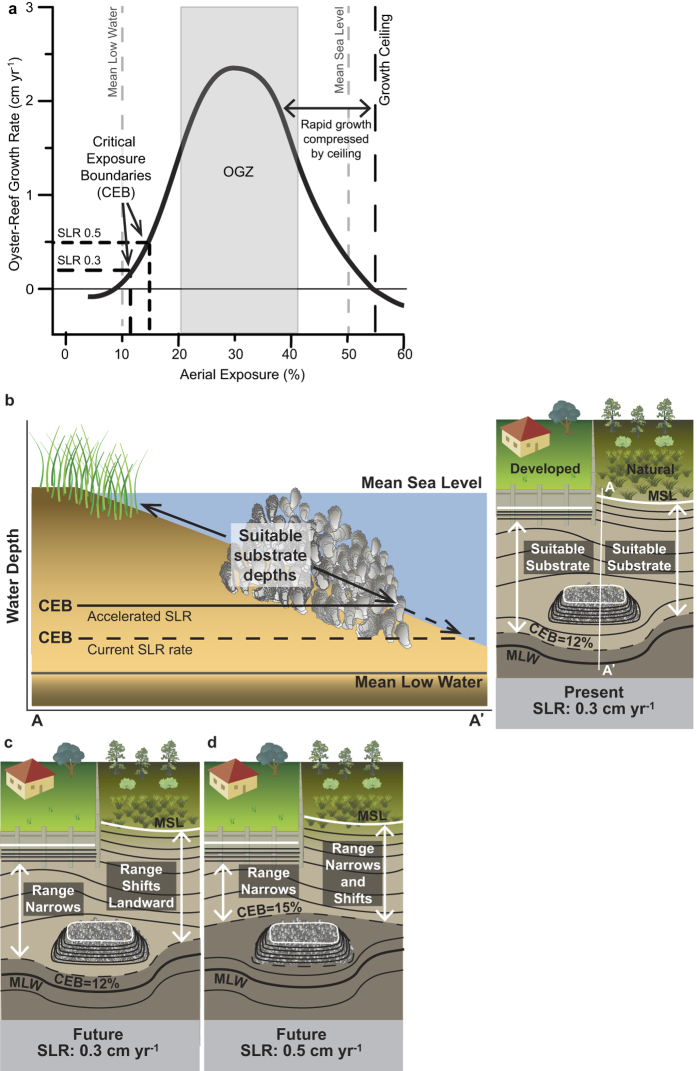
Modeling oyster-reef growth with aerial exposure and considering accelerations in SLR through time. (**a)** Greatest oyster-reef growth occurs at exposures between 20–40% (optimal-growth zone, OGZ) and returns to zero at 55% (growth ceiling) and 10% (mean low water). Critical-exposure boundaries (CEBs) represent reef growths in equilibrium with rates of local sea-level rise (0.3 cm yr^−1^ or 0.5 cm yr^−1^). (**b)** Suitable substrate (light shading), or range of viable habitat, for oyster reef development on developed and natural (retreating marsh) shorelines at initial time (Present) with a conceptual model of changing suitable substrate depths with reference to how differing rates of SLR will immediately change the CEB (SLR: 0.3 cm yr^−1^, SLR: 0.5 cm yr^−1^) along transect A-A′. For example, an oyster reef developing at 13% exposure can grow and persist at current rates of SLR, whereas an acceleration in SLR (0.5 cm yr^−1^) will result in the reef growth rate falling farther beneath the CEB (**a**) as sea levels rise at a faster rate than reef growth, leading to the reef’s eventual failure. (**c,d)** Future changes in suitable substrate (light shading) with CEBs considering SLR rates of 0.3 cm yr^−1^ (no change; (**c)**) and 0.5 cm yr^−1^ (accelerated; (**d)**). Future projections assume that nearshore sedimentation is not keeping pace with SLR. The range of suitable substrate depths will narrow against a developed shoreline, whereas natural shorelines will allow oyster reefs to shift shoreward (**c**). However, accelerations in SLR will raise the CEB, overall narrowing the range of suitable substrate depth regardless of the shoreface configuration (**d**). Incorporated symbols courtesy of the Integration and Application Network, University of Maryland Center for Environmental Science (ian.umces.edu/symbols/).

While rising sea level will shift the growth boundaries landward and to higher elevations, accelerations in SLR will exacerbate the loss of substrate elevations suitable for oyster reef growth. Similar to models of productivity in saltmarsh habitats^45^, our oyster-reef growth model reveals the rates of SLR for a given oyster reef to remain in equilibrium with rising water levels (Fig. 3a). At current rates of local SLR (~0.3 cm yr^−1^, ref. 46), the 12% exposure depth represents a critical-exposure boundary (CEB) where rates of reef growth and SLR are equal. At substrate depths above the CEB, oyster reefs will form and persist as a consequence of reduced stressors such as disease, predation, and sedimentation. However, an increase in the rate of SLR to 0.5 cm yr^−1^ (well within most predictions of SLR by 2100, ref. 47) may render substrates below 15% exposure unsuitable for intertidal oyster-reef habitat because below that level, oyster-reef accretion cannot keep pace with this SLR scenario (Fig. 3b). In contrast, the growth ceiling, while adjusting in elevation with SLR, will remain at the 55% exposure. Therefore, and most notably, this strong link between oyster growth and aerial exposure means accelerating SLR will reduce the estuarine area suitable for oyster reef occupation (Fig. 3c,d) between the shifting CEB (e.g., 12% to 15% exposure) and the constant growth ceiling (55%). The amount of oyster-reef habitat area lost locally further depends on nearshore sedimentation rates and changing bathymetry as the shoreface responds to SLR and fluctuations in sediment supply. Considering the model is crucial for newly forming oyster reefs (both natural and constructed), as we have witnessed failed reef growth below the CEB within the first year of construction^29^, making this boundary an immediate consideration for restoration efforts. Because our results suggest that oyster-reef growth in the intertidal zone is dependent upon percent aerial exposure, the range of suitable substrate depths (above the CEB) and the OGZ boundaries will likely expand in other estuaries of the U.S. with increasing tidal range as the intertidal zone is stretched across a greater depth spectrum (Supplementary Fig. 3). It also bears noting that the growth ceiling, depending upon oyster tolerance to desiccation and other stresses of exposure, may differ in warmer and colder latitudes, as more extreme temperatures could diminish the upper growth limit.

Older established oyster reefs that reached the growth ceiling are resilient to accelerating SLR because growth rates will increase on top of the reef as oysters exploit increased inundation time and subaqueous space. That increased productivity at the reef top could in turn lead to an increase in biogenic sediment flux to the reef base and enhance lateral and vertical accretion rates around the CEB. This resiliency is contingent upon limited disturbance; harvesting that lowers an oyster reef below the CEB will ultimately result in the loss of the habitat. Conservation efforts should limit harvesting practices from reducing oyster reef elevation below the OGZ to maximize the potential for rebound and to maintain optimal reef growth levels that would ensure the highest productivity of the fishery.

As development along low-elevation sheltered coastlines and rates of SLR continue to increase, so does our need for new decision-support tools that both reduce the risk of human societies to coastal hazards and maintain the vast natural capital that coastal habitats provide. In high salinity portions of estuaries, oyster-reef restoration in front of either saltmarsh shorelines or stabilization structures like riprap revetments will increase and help sustain ecosystem services, but only if restoration efforts consider the CEB and OGZ during project design, implementation, and future harvesting practices. Notably, the range of suitable substrate elevations for colonization, restoration, and maintenance of oysters and likely other intertidal foundation species is a moving and narrowing target with accelerating sea-level rise.

## Methods

We constructed reefs from 60 bushels of shucked oyster shell (cultch) formed into 3 × 5 × 0.15 m boxes in 1997, 2000, and 2011 on sandflats or adjacent to saltmarsh (*Spartina alterniflora* dominated; see Supplementary Fig. 1 and Table 1; refs 29,43). Constructed reefs are located within the Rachel Carson National Estuarine Research Reserve and are protected from harvesting. The natural reefs are located within the Rachel Carson National Estuarine Research Reserve and adjacent to Cape Lookout National Seashore and are not protected.

Ten-minute water-level data were obtained over the course of 6 months (June – December 2010) using HOBO® U20 Water Level Loggers (Onset Computer Corporation; ±0.3 cm accuracy) located in three areas of Middle Marsh. Loggers were placed in a stilling well (slotted PVC pipe) attached to rebar that was driven into the substrate to refusal (~3 m deep). Elevations were surveyed at both deployment and data collection, which occurred every month, with a Trimble^®^ RTK GPS. Pressure data were corrected for local fluctuations in barometric pressure using a fourth pressure sensor deployed on land, and water-levels were verified with independent field measurements obtained with a level measuring staff at time of deployment and readout. Survey data were used to transform the water-level data in to the North American Vertical Datum established in 1988 (NAVD88) with average vertical precisions of 1.5 cm. We divided the tidal data into 1-cm bins to ascertain mean percent aerial exposure at each elevation, which we used to convert all elevation measurements obtained from the oyster reefs to percent exposure.

Following previously established methods^41^, we measured cm-scale vertical growth rates across the entire surface of six sandflat reefs over a 2-year period. These reefs were chosen because they incorporated a wide range of intertidal elevations and were fully exposed during the most extreme spring low tides, which is necessary for our methods of measuring reef growth. Reefs were scanned using a Riegl three-dimensional LMSZ210ii terrestrial laser scanner in 2010 and 2012 and point clouds were processed to isolate ground returns using RiSCAN Pro software. We utilized Surfer 10 (Golden Software) to generate digital elevation models (1-cm cell size) of the six sandflat reefs from 600,000 to 1,000,000 laser returns (number depends on reef size) spaced <1 cm apart. Elevation changes >1.4 cm are resolvable with this method. The 2010 reef grid-cell elevations were subtracted from 2012 counterparts (>500,000 observations per reef) to obtain elevation changes between measurements. This allowed us to create a table of XYZ and elevation change (2010 value for Z), which we sorted by descending 2010 elevation values. Those data were separated into 2-cm elevation bins (2010 elevations) and mean elevation change between 2010 and 2012 for each bin were calculated (i.e., mean vertical accretion rate for every 2-cm change in reef elevation across the entire reef surface). An overall mean vertical accretion rate among reefs was then calculated for each 2-cm elevation bin. Reef MF4-2000 was excluded from the overall mean because it showed signs of significant harvesting between our 2-yr time step (even though the reef was protected), and this was supported by laser-scan data and field observations.

Recently constructed reefs (2011) were placed on sandflats at approximate substrate elevations of −0.9 m, −0.75 m, −0.6 m, and −0.5 m NAVD88 (exposure range: 0.01–18%, see refs 24,30). To assess the growth of recently-constructed (2011) reefs over their lifetimes, we surveyed a grid across each reef using the RTK-GPS at 0.25-m horizontal intervals. Reefs were surveyed in the fall and winter of 2011, spring of 2013, and spring of 2014. The highest 10% of points within each grid were averaged and designated as the elevation of the reef top. For each reef, we subtracted top elevations between the longest available time step and then normalized by the time interval. For comparison, reef accretions were averaged after binning by original cultch surface exposures: 0–15%, 15–30%, and >30%.

To ascertain size-abundance patterns at different aerial exposures we measured oyster density and oyster-shell height across intertidal elevations. We randomly placed 0.25-m^2^ quadrats at varying elevations (surveyed using the Trimble^®^ GPS) on decade-old reefs to obtain 2–3 quadrat samples per reef (*N *= 22) for density and SH. To sample natural reefs (*N *= 7), parallel transects, from reef crest to base, were placed 1 meter apart, and one randomly-placed quadrat was sampled along each transect. Samples within reefs were not pooled because they were collected from areas with different exposure conditions, and our primary interest was to determine if exposure regulates reef dynamics. Oyster-reef material was sampled to a depth of approximately 15 cm (or to the depth where sediment was anoxic) and the number and shell height of live oysters was quantified in the field. Samples were broken into 4 different aerial exposure bins: less than mean low water (<MLW), from MLW to 20% aerial exposure (MLW), from 20% to 40% aerial exposure or the optimal-growth zone (OGZ), and greater than 40% aerial exposure (>OGZ). Adult-oyster densities (oysters >2.5 cm long) were then analyzed among aerial exposure bins and between natural and constructed reefs using a two-way analysis of variance (ANOVA). We used a *post-hoc* Tukey Honest Significant Difference (HSD) test (α = 0.05) to determine density differences among exposure zones and between reef types.

The tidal-growth model (Fig. 3a) was developed using water-level data and the oyster-reef-growth curve (Fig. 2a). Critical boundaries were defined as the elevation where reef growth equaled the current or forecasted rate of sea-level rise. NOAA tide data from Fort Pulaski, Georgia (Station ID: 8670870) were used to extrapolate the model into a larger tidal range (2.25 m)(Supplementary Fig. 3). Tidal elevations were transformed to aerial exposure and indexed to corresponding reef-growth rates using the North Carolina growth curve.

## Additional Information

**How to cite this article**: Ridge, J. T. *et al.* Maximizing oyster-reef growth supports green infrastructure with accelerating sea-level rise. *Sci. Rep.* **5**, 14785; doi: 10.1038/srep14785 (2015).

## Supplementary Material

Supplementary Information

Supplementary Dataset 1
