# Supplementary material for: Maximizing oyster-reef growth supports green infrastructure with accelerating sea-level rise
Source: Sci Rep. 2015 Oct 7;5:14785. doi: 10.1038/srep14785 (PMC4595829; doi:10.1038/srep14785)

**Maximizing oyster-reef growth supports green infrastructure with accelerating sea-level rise**

**Authors:** Justin T. Ridge^1,*^, Antonio B. Rodriguez^1^, F. Joel Fodrie^1^, Niels L. Lindquist^1^, Michelle C. Brodeur^1^, Sara E. Coleman^1,†^, Jonathan H. Grabowski^2^, and Ethan J. Theuerkauf^1^

**Supplementary Materials**

**
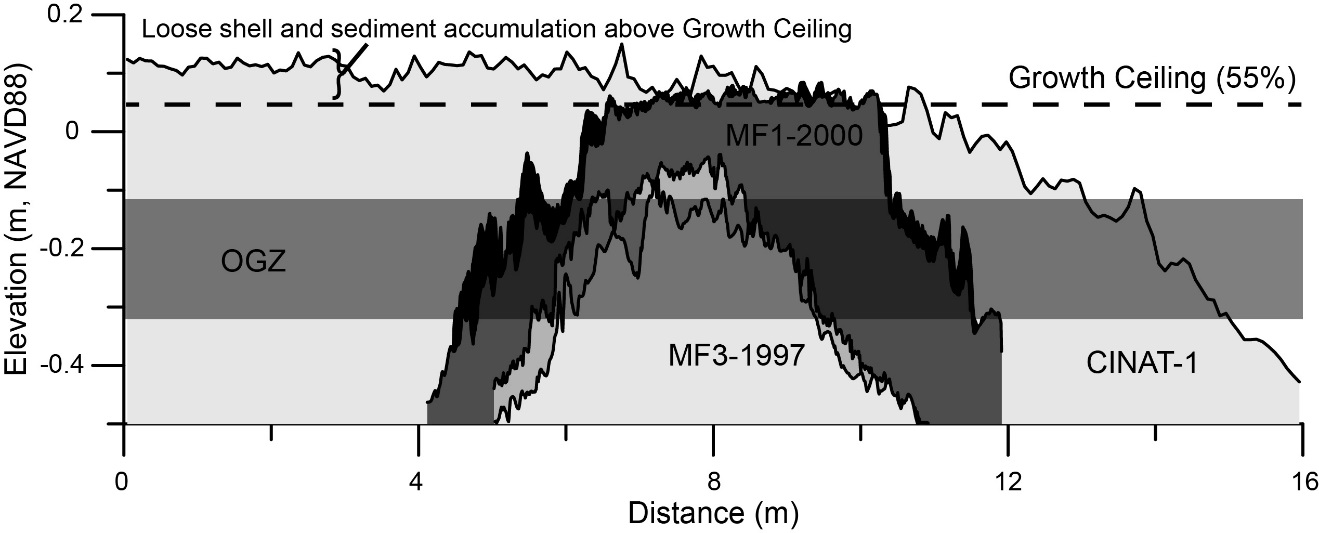
**

**Supplementary Figure 1 | Example profiles from three study reefs.** Reefs form plateaus around 55% aerial exposure. The optimal-growth zone (OGZ), occurring between 20-40% aerial exposure is highlighted. Profiles from MF3-1997 and MF1-2000 are from reef scans taken in 2010 (lighter) and 2012 (darker). CINAT-1 is a natural fringing reef.

**
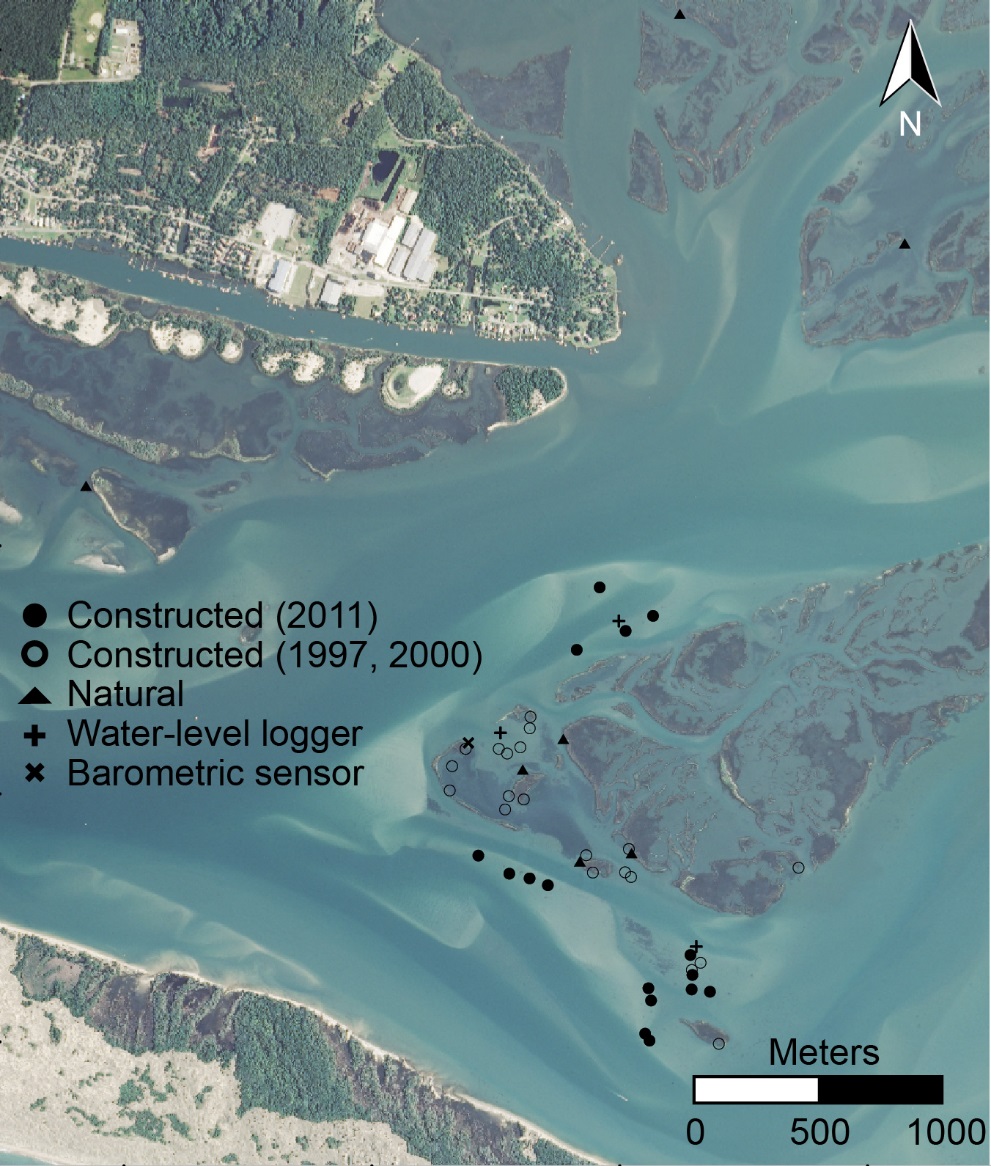
**

**Supplementary Figure 2** **|** **Map of study area in Back Sound, North Carolina**. Sampled reefs and locations of water-level loggers are indicated. Map created with Surfer® 11 (Golden Software) and Adobe® Illustrator (Adobe Systems) using aerial imagery from the United States Department of Agriculture National Agriculture Imagery Program.


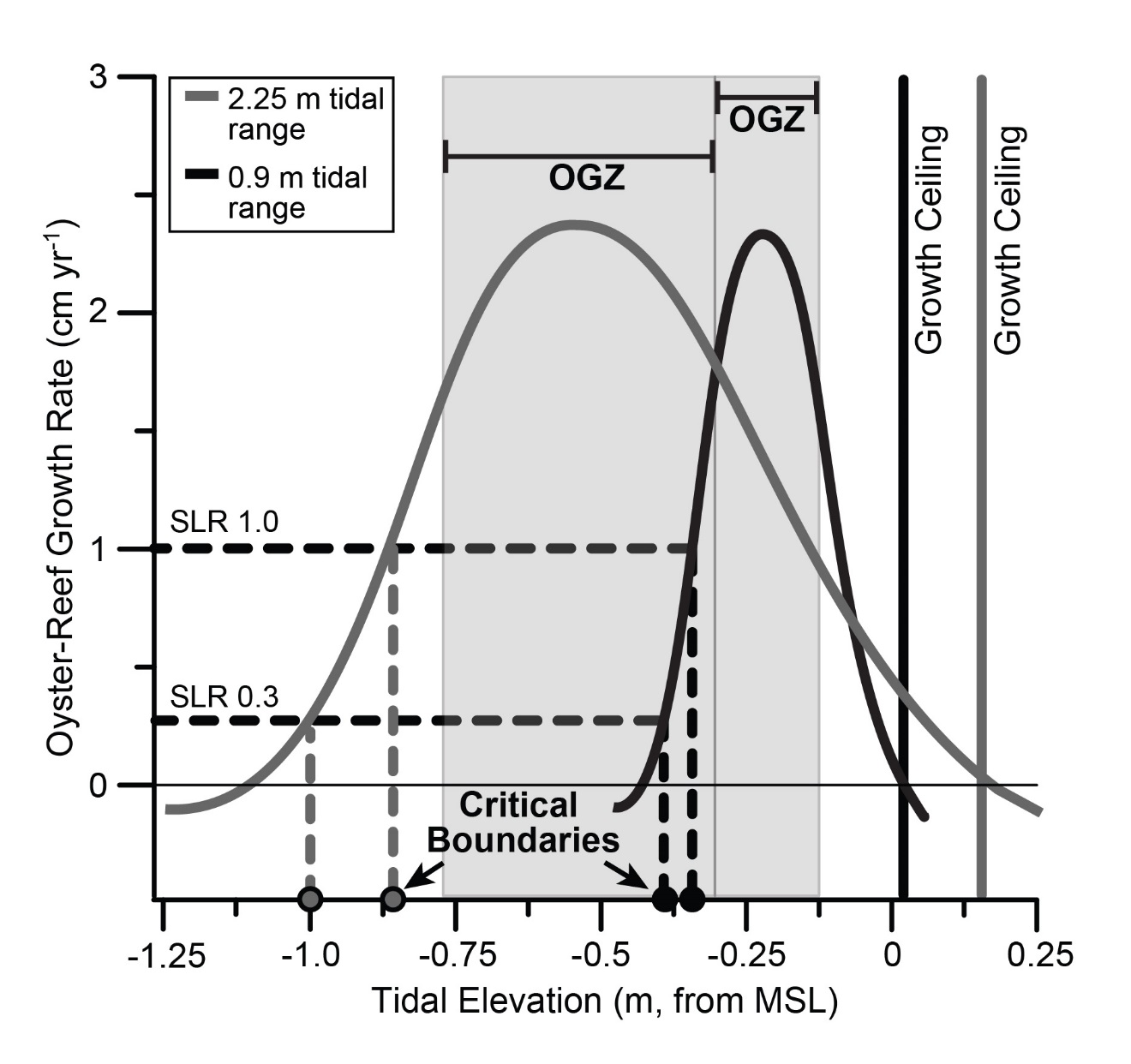


**Supplementary Figure 3** **| Expanded growth-tidal height model**. Including a greater tidal range (2.25 m), model illustrates a larger OGZ (bounded by 20-40% aerial exposure) but greater loss of suitable substrate elevations above the critical exposure boundary with a SLR-rate acceleration from 0.3 to 1.0 cm yr^-1^. Growth rates are approximate.

**Supplementary Table 1** **|** **Sample breakdown of study reefs.**

Reef locations (UTM), type, and sampling conducted of all oyster reefs within the study.


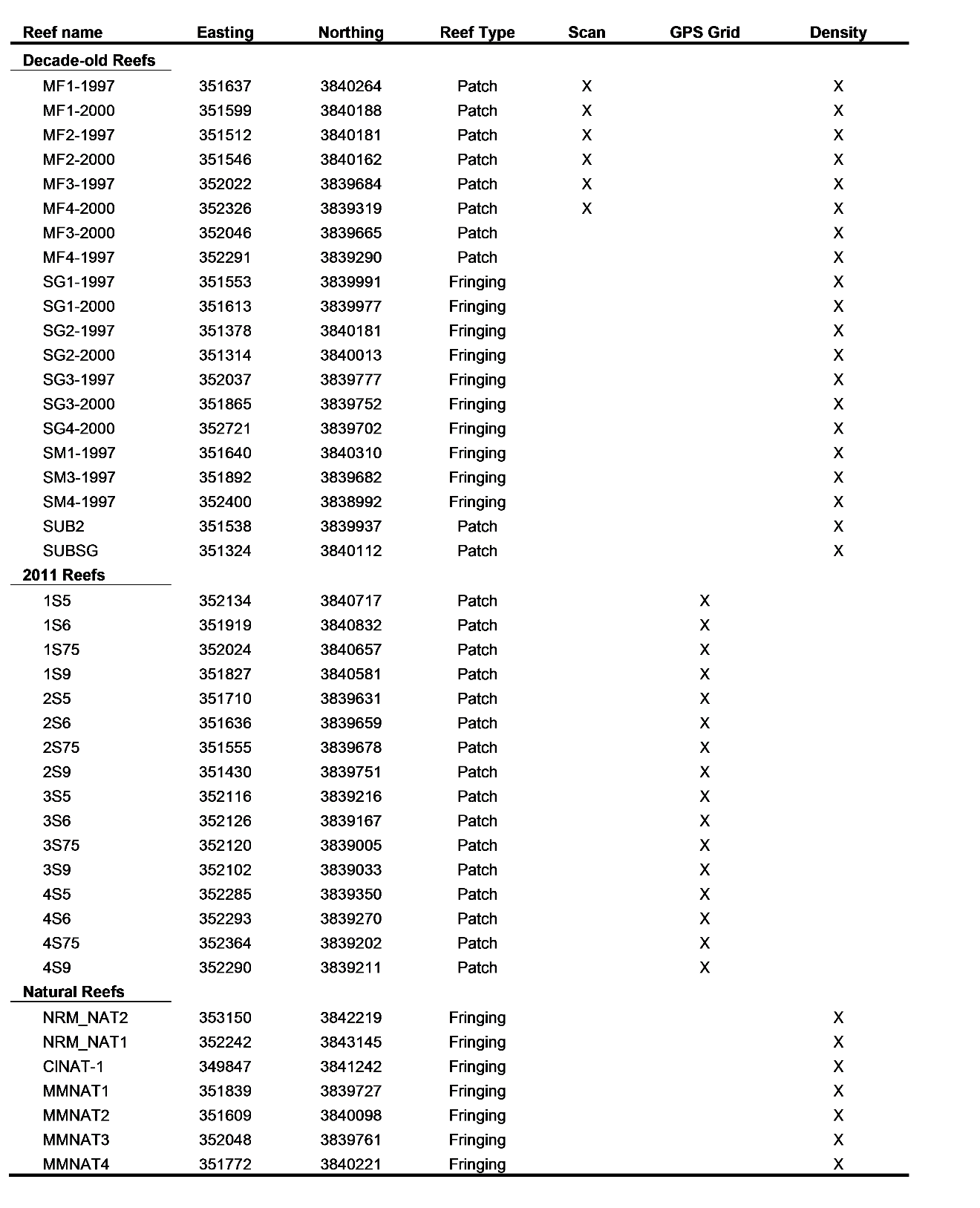

Supplement: Supplementary Information [file srep14785-s1.doc]
